# Editorial: Cardiovascular remodeling

**DOI:** 10.3389/fphar.2023.1252598

**Published:** 2023-07-24

**Authors:** Yan Yang, Yi Zhang, Gui-Rong Li, Ming Lei

**Affiliations:** ^1^ Key Laboratory of Medical Electrophysiology of Ministry of Education and Medical Electrophysiological Key Laboratory of Sichuan Province, Institute of Cardiovascular Research, Southwest Medical University, Luzhou, China; ^2^ Department of Physiology, Hebei Medical University, Shijiazhuang, China; ^3^ Nanjing Amazigh Pharma Ltd., Nanjing, China; ^4^ Department of Pharmacology, University of Oxford, Oxford, United Kingdom

**Keywords:** cardiovascular diseases (CVDs), cardiovascular remodeling, genetic studies, dysfunction, drug therapy

## Abstract

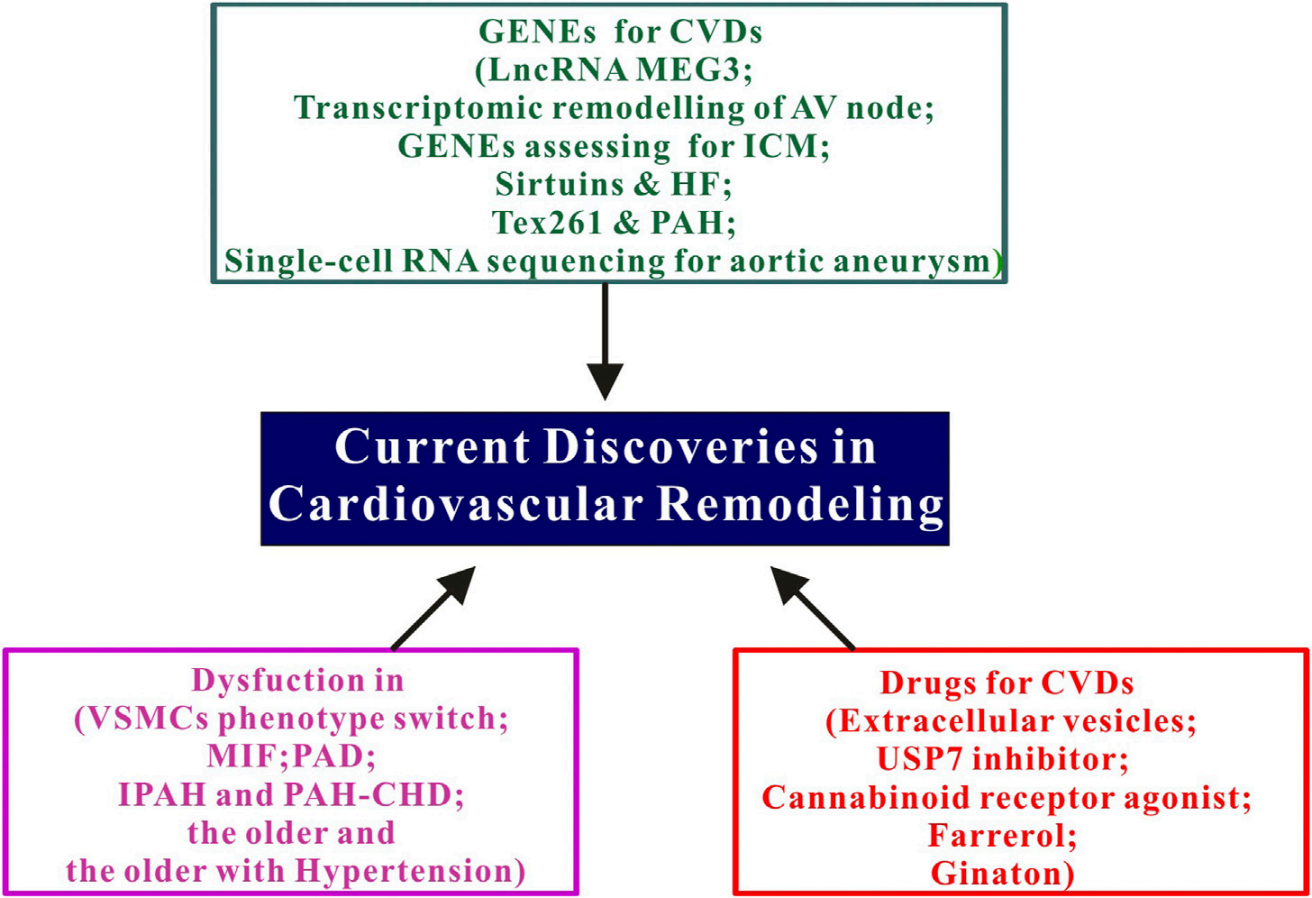

## 1 Introduction

Cardiovascular diseases (CVDs) remain as the leading cause of death worldwide, highlighting the need for new therapeutic approaches. A significant pathogenic factor contributing to the development of CVDs is cardiovascular remodeling. As such, this Research Topic aims to explore and evaluate recent progress in molecular mechanisms, drug therapy, and innovative technologies, with the ultimate goal of enriching our understanding and management of cardiovascular remodeling. The Research Topic contains three integrated sections, as summarized in the Graphical Abstract and the subsequent parts.

## 2 Genetic studies in relation to cardiovascular disease

The significance of the maternally expressed gene 3 (MEG 3) is discussed in a review article Li et al. MEG3 is involved in the processes of various human diseases. P53 and PI3K/Akt are important pathways for MEG3 to participate in the regulation of apoptosis. MEG3 binds to miRNA directly or competitively and is involved in apoptosis, inflammation, oxidative stress, endoplasmic reticulum stress and epithelial-mesenchyme transition. LncRNA MEG3 is mainly involved in malignant tumors, metabolic diseases, immune system diseases, cardiovascular and cerebrovascular diseases. LncRNA MEG3 has a variety of pathological effects in cardiomyocytes, fibroblasts and endothelial cells, and has a great potential for clinical application in the prevention and treatment of atherosclerosis, myocardial ischemia-reperfusion injury, hypertension and heart failure (HF). A study conducted by Wilson et al. aimed to determine the underlying cause of atrioventricular (AV) node dysfunction in HF by analyzing changes in the transcriptome of the entire AV node. The research utilized the mouse transverse aortic constriction model, which induces HF through pressure overload. Functional changes were assessed through electrocardiography and echocardiography, while the transcriptome of the AV node was quantified using RNAseq. The findings indicate that AV node dysfunction in HF is attributed to inflammation and a pervasive transcriptional remodeling within the AV node. Another crucial point to consider is the significance of the SIRT1 pathway in the pathophysiology of myocardial remodeling and HF, as demonstrated by the Wang et al. Sirt1, a nicotinamide adenine dinucleotide + -dependent deacetylase, emerges as a fundamental player in the pathophysiology of myocardial remodeling and HF. Its involvement in transcriptional regulation, energy metabolism, cell survival, DNA repair, inflammation, and circadian regulation positions it as a pivotal molecule for investigation. The existing literature indicates substantial advancements in understanding the role of the SIRT1 pathway in the pathophysiological mechanisms underlying myocardial remodeling and HF. The original research articles by Lu et al. primarily focused on the discovery of several newly identified therapeutic targets. They developed a risk model based on the progression of necrosis in order to identify the genes associated with this process. The objective of their study was to identify potential therapeutic agents and evaluate the progression and prognosis of ischemic cardiomyopathy (ICM) by screening for necroptosis-related genes and constructing a risk score. The findings of their study provide important insights into the significance of six necroptosis-related signature genes (STAT4, TNFSF10, CHMP5, CHMP18, JAK1, and CFLAR) in assessing the progression and prognosis of ICM. These signature genes have significant clinical value and could potentially serve as targets for enhancing cardiovascular remodeling. Tex261 is a protein-coding gene whose functional enrichment node includes the transporter activity of COP II. Chen et al. conducted a study on the correlation between Text6 and pulmonary hypertension (PAH) on the basis of multi-omics sequencing to screen target proteins. Tex261 has been found to be involved in COPII and apoptosis regulation. Hypoxia inhibits Tex261 expression by activating HIF-1α. Downregulation of Tex261 promoted Ndrg1 and inhibited AKT activity, which in turn inhibited Sec23 activity, leading to cell proliferation and vascular remodeling. The increase of Tex261 has certain preventive and therapeutic effects on PAH in rats. These results provide a basis for exploring whether Tex261 can be used as a target for prevention and treatment of PAH, and provide a new possibility for clinical diagnosis and treatment. These positive effects are mediated through the NF-κB signaling pathway. Most worth mentioning in this Research Topic is “*Comparative analysis of thoracic and abdominal aortic aneurysm across segment and species*” (Wu et al.). Single-cell RNA sequencing analysis was used to compare the differences in the cell populations of mouse and human aneurysms in the abdominal aorta and thoracic aorta, revealing the similarities and differences in the changes in the cell type components of human and mouse, and identifying the well-preserved SMCs and macrophage subpopulations. In addition, the analysis results indicated that the biological functions of thoracic and abdominal aortic aneurysms are different, and the underlying pathogenic genes are also different. This study defines the pattern of aortic aneurysms, and lays the foundation for future research into the potential of subgroups or marker genes as therapeutic targets.

## 3 Dysfunction and novel mechanisms in cardiovascular diseases

A bibliometric analysis (Han et al.) on the phenotype switch of vascular smooth muscle cells (VSMCPS) has garnered significant interest. To create a visual representation of the knowledge landscape in VSMCPS research, a bibliometric analysis was conducted. The Web of Science Core Research Topic Database (SCI-EXPANDED) was utilized to search for literature on VSMCPS from 1999 to 2021. Bibliometric tools such as VOSviewer and CiteSpace were employed. The study identified the most productive researchers, journals, institutions, and countries in this field. Furthermore, trends, hot topics, and knowledge networks were analyzed and visualized, demonstrating an increasing number of annual publications in the field of VSMCPS. The primary research topics include “vascular remodeling,” “atherosclerosis,” “intimal neogenesis,” “hypertension,” and “inflammation.” The article “*Treatment of myocardial interstitial fibrosis in pathological myocardial hypertrophy*” (Zhu et al.) provides a comprehensive overview of the current understanding of the mechanisms and negative consequences associated with myocardial interstitial fibrosis (MIF) in pathological cardiac hypertrophy. Pathological myocardial hypertrophy, which can arise from various diseases, often coincides with the occurrence of MIF. This condition involves the formation of diffuse, patchy lesions comprising interstitial microscars and the deposition of collagen fibers around blood vessels. This article also explores the potential use of circulating and cardiovascular magnetic resonance (CMR) imaging biomarkers to identify MIF lesions, while also discussing existing and potential future therapeutic strategies. In a study conducted by Zhu et al., researchers investigated the primary function of the right ventricle (RV) and its subsequent response to targeted therapies in patients diagnosed with idiopathic pulmonary arterial hypertension (IPAH) and pulmonary arterial hypertension associated with congenital heart disease (PAH-CHD). The findings revealed that patients with IPAH had lower baseline RV function, a less favorable prognosis, and a limited response to targeted treatment compared to those with PAH-CHD. Multiple risk factors, such as smoking, dyslipidemia, diabetes, and hypertension, have been identified as contributors to the development of peripheral vascular diseases (PAD). The article titled “*Peripheral vascular remodeling during ischemia*” (Lin et al.) discussed in depth the functional changes observed in peripheral arterial cells during ischemic conditions and explores the underlying mechanisms responsible for the pathogenesis of PAD. Additionally, the article provides an overview of the current advancements in clinical treatments and potential therapeutic approaches for PAD, while shedding light on future perspectives in this field. The thromboxane A2 (TXA2) receptor (TP) is a member of the G protein-coupled receptors (GPCRs) family. The activation of TP receptor ultimately depends on the availability of a specific G protein. Zhang et al. discovered that the TXA2-TP prostaglandin signaling pathway is impaired in elderly rats. Their research demonstrated that the contraction of mesenteric arterioles induced by thromboxane was diminished in the older and the older hypertensive rats. This suggests a weakened TXA2-TP prostaglandin signaling pathway in the older. These findings complement the understanding of the mechanisms underlying changes in vascular TXA2-TP signaling during vascular remodeling associated with aging.

## 3 Enhancement of cardiovascular health through drug therapy

One article (Cheng et al.) delves into the topic of extracellular vesicles (EVs)—bilayer lipid nanoscale vesicles—that are released into the extracellular space by eukaryotic cells. These vesicles act as transmitters of biological information and play a vital role in intercellular communication. This article expands upon the aforementioned concept and discusses the advancements achieved in pharmacology and engineering for the purpose of targeting EVs to facilitate cardiac repair following a myocardial infarction (MI). Furthermore, this article delves into the dual functionality of the small molecule transmitters contained within EVs, with regards to their influence on the process of post-infarction cardiac remodeling. Ubiquitin specific protease 7 (USP7) plays a crucial role in various biological processes. In Gu et al.’s study, it was found that the expression of USP7 was elevated in both mice and patients with HF induced by Ang II. Additionally, the researchers observed that the inhibitor p22077 was able to mitigate cardiac hypertrophy, cardiac fibrosis, inflammation, and oxidative stress by inhibiting the AKT/ERK, TGFβ/SMAD2, NFκB/NLRP3, and NOX2/NOX4 signaling pathways. These findings indicate that USP7 could be a potential therapeutic target for managing hypertrophy remodeling and HF. It was previously hypothesized that cannabinoid receptors (CBR) possess cardioprotective properties. CB13, a compound that activates both cannabinoid receptors, has been found to stimulate AMPK signaling in ventricular cardiomyocytes. The effects of CB13 on atrial myocyte expansion and mitochondrial function in neonatal atrial rat cardiomyocytes (NRAM) stimulated by angiotensin II (AngII) were investigated in a study conducted by Altieri et al. Their findings provide further evidence that activation of CBR promotes AMPK activation in the atria, ultimately preventing myocyte enlargement, mitochondrial depolarization, and destabilization of Cx43. It has been speculated that Farrerol may have a significant role in reducing heart hypertrophy and remodeling. He et al. conducted a study to assess the role of Farrerol in cardiac remodeling. Firstly, they established a model of myocardial remodeling, and subsequently administered Farrerol through intraperitoneal injection to evaluate its effects. The results revealed that Farrerol effectively inhibited the Ang II-induced hypertrophy of cardiomyocytes, oxidative stress levels, as well as the proliferation and migration of fibroblasts. These findings suggest that Farrerol holds promise as a potential candidate for the treatment of myocardial remodeling. In addition, in traditional Chinese medicine related studies, Wang et al. demonstrated that the use of Ginaton, a natural extract derived from Ginkgo biloba, effectively inhibits the activation of M1 phenotype macrophages induced by Ang II. This treatment also prevents macrophage adhesion and attenuates the inflammatory response, thereby leading to the alleviation of hypertension and cardiac remodeling.

In summary, this topic gives us a glimpse into the ongoing research progress on cardiovascular remodeling. It is expected that these efforts will aid in future research endeavors aimed at comprehending the fundamental mechanisms of cardiovascular disease and pave the way for effective therapeutic strategies.

